# Autoimmune Oral Mucosal Diseases in Older Patients: An Overview of Early Recognition and Management in the Primary Care Setting

**DOI:** 10.3390/geriatrics11040102

**Published:** 2026-08-11

**Authors:** Anahita Gupta, Donna A. Culton

**Affiliations:** 1 School of Medicine, University of North Carolina, Chapel Hill, NC 27599, USA; 2 Department of Dermatology, University of North Carolina, Chapel Hill, NC 27599, USA; donna_culton@med.unc.edu

**Keywords:** pemphigus vulgaris, mucous membrane pemphigoid, oral lichen planus, autoimmune oral mucosal disease

## Abstract

Autoimmune oral mucosal diseases, including pemphigus vulgaris, mucous membrane pemphigoid, and oral lichen planus, are important yet frequently underrecognized causes of morbidity in older adults. These conditions often initially present with nonspecific symptoms such as persistent oral pain, erosions, ulcerations, or gingival inflammation, contributing to delays in diagnosis and treatment. Primary care clinicians and geriatricians are uniquely positioned to identify these diseases early and facilitate timely referral for diagnostic confirmation and initiation of therapy. This review provides a practical overview of the clinical presentation, diagnostic evaluation, and initial management of pemphigus vulgaris, mucous membrane pemphigoid, and oral lichen planus and highlights features that should raise suspicion for an autoimmune oral process. In addition to disease-specific therapies, supportive care measures including pain control, oral hygiene counseling, nutritional support, and mental health screening are essential to reducing morbidity and improving quality of life. Special consideration should be given to challenges disproportionately affecting older adults, including frailty, polypharmacy, and social barriers to care. Through timely recognition and a multidisciplinary, patient-centered approach, clinicians can improve outcomes and support the quality of life of this patient population.

## 1. Introduction

Autoimmune conditions affecting the oral mucosa, including pemphigus vulgaris, mucous membrane pemphigoid, and oral lichen planus, are important yet frequently underdiagnosed causes of morbidity, particularly among older adults. These disorders often initially present with nonspecific oral symptoms such as persistent erosions or ulcers, gingival erythema and inflammation, and/or mucosal pain, which may be mistaken for more common benign conditions [1,2,3]. As a result, long delays in diagnosis are common, and many patients experience prolonged symptoms and diminished quality of life before receiving appropriate evaluation and treatment [4,5,6].

Primary care clinicians and geriatricians are uniquely positioned to identify these conditions early, as they are often the first and most frequent point of contact for patients. Recognizing key clinical features, such as chronic, nonhealing erosions, mucosal fragility, and inflamed mucosa, is critical to initiating work-up for an underlying autoimmune process. Early identification facilitates timely referral to medical specialties for diagnostic confirmation and initiation of appropriate therapy, which can significantly reduce morbidity and improve long-term quality of life. In addition to early recognition, initial management in the primary care setting plays an important role in improving patient outcomes. Symptom-directed interventions, including pain control, oral hygiene guidance, and nutritional support, can help maintain oral intake and quality of life while patients await specialty care. Primary care providers also play a critical role in evaluating and supporting mental health, as these autoimmune conditions can be associated with increased rates of anxiety and depression [7,8,9,10]. These measures are particularly important in older adults, who are more vulnerable to complications such as weight loss, frailty, and functional decline, as well as face increased social barriers to care [2,11].

This narrative review aims to provide primary care clinicians and geriatric providers with practical guidance on the early recognition, initial management, and appropriate referral of patients with pemphigus vulgaris, mucous membrane pemphigoid, and oral lichen planus. By promoting timely diagnosis and a supportive, patient-centered approach, clinicians can play a pivotal role in reducing disease burden and improving outcomes in this population.

## 2. Pemphigus Vulgaris

**Figure 1:**
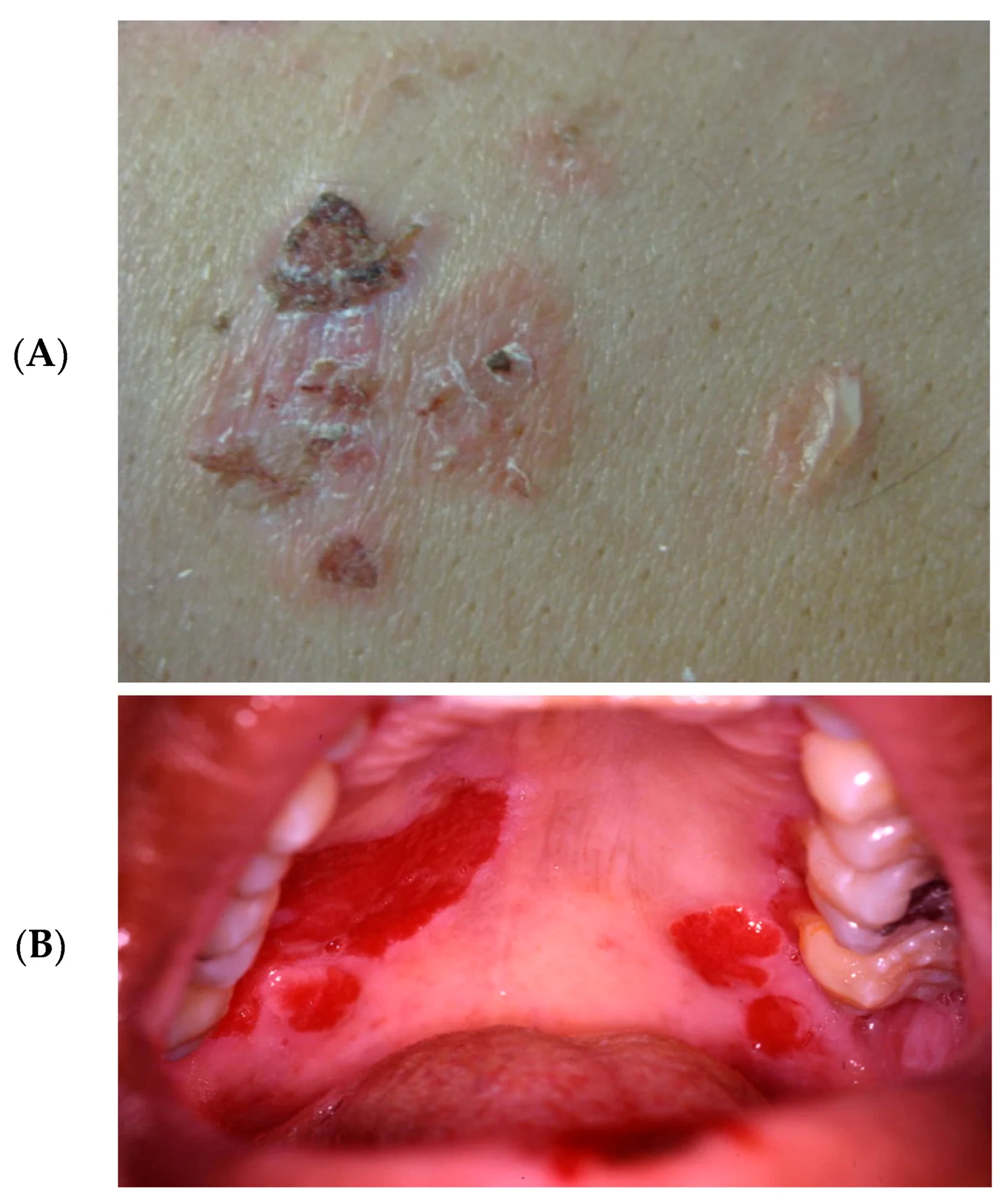
Pemphigus vulgaris. (**A**) Flaccid vesicles and bullae that rupture, leaving erosions of the skin. (**B**) Erosions of the hard palate.

Pemphigus vulgaris (PV) is a chronic autoimmune blistering condition that affects the oral mucosa in older adults. PV classically presents with fragile, thin-walled cutaneous blisters that rupture easily, leaving behind painful erosions with poor healing and the potential for secondary infection (Figure 1A) [1]. Interestingly, oral lesions are the most common manifestation of PV, with 90% of patients developing oral lesions over the course of their disease. Oral lesions also provide an important diagnostic clue, seeing as oral involvement is the first sign of PV in 50–70% of patients [12]. These lesions manifest as irregularly shaped erosions of variable sizes on the buccal, palatine, labial, and gingival mucosae (Figure 1B) [13]. Symptomology of these lesions varies widely, from being asymptomatic to causing debilitating pain, and can make it difficult for patients to eat and maintain their nutrition. In ~40% of patients, mucosal lesions may be the only manifestation of PV (so-called mucosal-dominant PV), with oral mucosa being the most common mucosal site involved, though other mucosal sites may be involved, including the laryngeal, esophageal, genital, and perianal surfaces [5,12,14]. In 60% of patients, blisters extend beyond the mucosal tissues to affect the cutaneous surfaces (mucocutaneous PV). Lesions on the skin present on the trunk, groin, axilla, scalp, and extremities, typically sparing the palms and soles [12].

PV is associated with an increased risk of overall mortality due to the effects of open wounds and treatment [14]. Because patients can present with large open wounds, particularly those with cutaneous involvement, they are at a much greater risk of infection and sepsis. This is amplified by the fact that treatments of PV typically involve immunosuppressive therapy, increasing patients’ risk of contracting new infections and having poor recovery [15,16]. Aside from mortality, patients often face downstream consequences from painful oral erosions, including difficulty eating and performing oral hygiene, as well as possible dysphagia if there is pharyngeal or esophageal involvement. This can eventually manifest as malnutrition, weight loss, tooth decay, and gum disease—all of which worsen patients’ overall physical and mental health [17]. Because of these risks, older adults and immunocompromised individuals with PV typically have a worse prognosis [11].

### 2.1. Epidemiology and Pathophysiology

PV is caused by IgG autoantibodies directed against desmogleins (notably desmogleins 3 and 1), which are transmembrane cadherin-type glycoproteins that maintain cell-to-cell adhesion [14,18]. Autoantibody binding disrupts desmoglein binding and desmosomal integrity, leading to loss of keratinocyte adhesion (acantholysis). This process results in intraepithelial blister formation and the characteristic flaccid, fragile bullae and erosions observed in PV.

Although epidemiological data for PV is limited given its relative rarity, PV is estimated to have an incidence of 0.1 to 0.5 per 100,000 people [1,19]. Its incidence may be higher based on ethnic and geographical factors, with higher rates reported amongst people of Ashkenazi Jewish, Middle Eastern, and Southeast European descent [1]. Men and women are typically equally affected, and older adults tend to be more impacted by PV, often presenting in the fifth or sixth decade of life [1,14]. Studies have also shown a genetic component of PV; although it does not seem to be inherited in a Mendelian pattern, there are certain genetic combinations that may increase susceptibility to PV. Specifically, certain human leukocyte antigen (HLA) class II alleles have been associated with increased rates of PV, indicating that the pathogenesis of PV may be multifactorial and involve both genetic and environmental triggers [20].

PV can also be induced by certain drugs, though drug-induced PV and idiopathic PV cannot be differentiated based on clinical exam or laboratory testing. The most common drugs that trigger PV possess thiol or phenol groups and include penicillamine, captopril, and tiopronin [11,19,21,22,23]. If drug-induced PV is suspected, the drug should be stopped immediately, though standard-of-care treatment should not be delayed.

### 2.2. Diagnosis and Work-Up

The diagnosis of PV is made through a combination of clinical history, clinical exam findings, histopathology, and immunopathology. A thorough clinical history is essential in patients with suspected PV, as mucosal lesions often precede cutaneous involvement and may initially be misdiagnosed. Clinicians should inquire about the characteristics of the lesions, particularly if they are erosive, painful, or heal slowly. Another important consideration is whether there is mucosal involvement outside the oral cavity, and clinicians should evaluate for dysphagia, odynophagia, voice hoarseness, and dyspareunia when assessing for other sites of mucosal involvement, as well as inquire about cutaneous findings. A thorough review of all medications should also be conducted to identify whether the patient recently started a drug that could induce PV. This history should be coupled with a thorough physical exam of the oral cavity. Bullae and vesicles are rarely seen on the mucosal surfaces, as lesions tend to rupture easily; thus, erosions are more typically seen with lesions on the buccal or palatine mucosa, as well as on the gingiva or labial mucosa [12]. A characteristic exam finding of PV is Nikolsky’s sign, where light pressure near a lesion causes epithelial detachment; although this is not pathognomonic for PV, it raises clinical suspicion for this diagnosis.

Diagnosis relies on histopathological evaluation, including biopsies for hematoxylin and eosin (H & E) or direct immunofluorescence (DIF), and serological studies, including indirect immunofluorescence (IIF) or ELISA. H & E would reveal suprabasilar acantholysis, while DIF would demonstrate IgG and complement deposition in the intercellular spaces in a netlike pattern [1,11]. Serology would reveal autoantibodies against desmoglein 3 and in some cases against desmoglein 1 [11]. Broad laboratory work-up is not helpful for diagnosis but may guide selection of systemic treatment [12]. Other relevant testing to consider at baseline includes a DEXA scan (in preparation of potentially long-term steroid treatment), as well as mental health screenings like the PHQ-9 or GAD-7 to evaluate quality of life [5].

### 2.3. Treatment

Given that PV is an autoimmune condition, treatment typically requires a balance between suppressing the immune system and preventing treatment-related adverse effects [21]. First-line treatment is systemic corticosteroids given the rapidity of onset and accessibility/affordability, with prednisone being the most commonly used steroid. The effect of steroids on PV is rapid; improvements in existing lesions may occur within a few days, and new lesions may cease to appear after 2–3 weeks of treatment, though relatively high doses of 1 mg/kg/day are needed to achieve this response [12]. Complete re-epithelization can take up to 2 months. Once re-epithelization has occurred and new lesions are not appearing, patients can be tapered off the steroid and monitored for relapses, which are likely to occur in the absence of accompanying adjuvant therapy. The side effects of steroids are well-known and include arterial hypertension, diabetes mellitus, osteoporosis, glaucoma, and increased risk of infections [12]. These side effects should be carefully considered based on the patient’s profile when choosing treatment.

Adjuvant therapies are initiated early in the course of treatment to facilitate a steroid-sparing effect [5,12]. Rituximab is considered first-line adjuvant therapy, with studies demonstrating that rituximab can lead to sustained remission in patients, particularly if initiated within 6 months of diagnosis, with superiority over other immunosuppressive agents such as mycophenolate [24,25]. Serious adverse effects are rare but can include severe infusion reactions, neutropenia, severe infections, and very rarely hypogammaglobinemia [12]. Other adjuvant therapy options include azathioprine, mycophenolate mofetil, intravenous immunoglobulin, and cyclophosphamide [21]. Each therapy has a different side-effect profile, which should be carefully considered based on individual patients’ risk factors before use.

## 3. Mucous Membrane Pemphigoid

**Figure 2:**
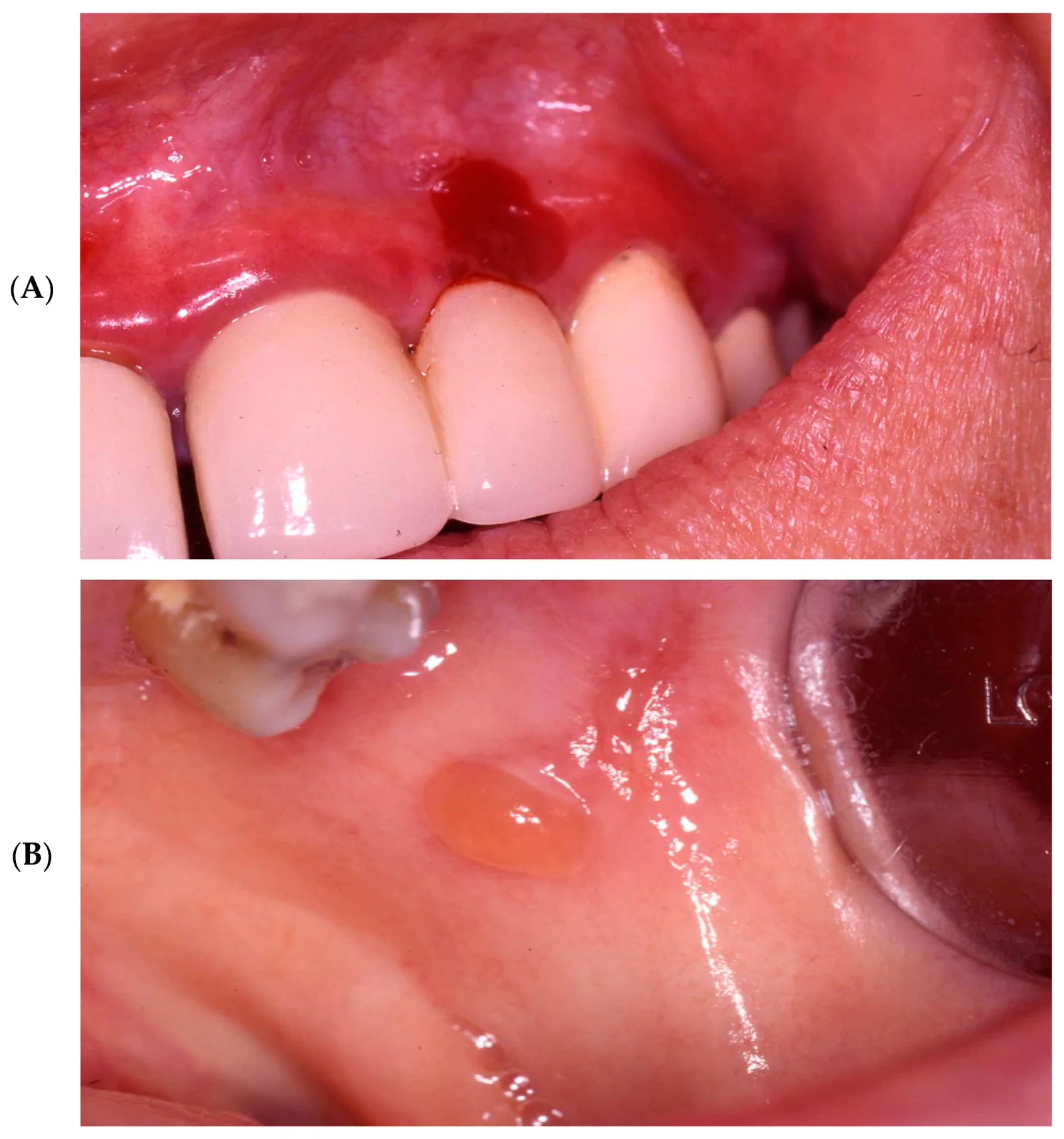
Mucous membrane pemphigoid. (**A**) Erythema and edema of the maxillary facial gingiva. (**B**) Tense vesicle of the buccal mucosa.

Mucous membrane pemphigoid (MMP) is an umbrella term that comprises subepidermal blistering diseases characterized by predominantly mucosal involvement. MMP typically affects the oral mucosa, with desquamative gingivitis being a common presentation that manifests as erythema and edema of the gingiva that clinically mimic periodontal disease (Figure 2A) [2,17]. Other clinical features include tense vesicles (Figure 2B) that often rupture, resulting in irregularly shaped ulcerations with possible overlying pseudomembrane formation (moist fibrinous covering that forms over oral ulcers) [26]. Lesions can occur anywhere in the oral cavity but most often occur on the buccal, gingival, and palatine mucosae; labial involvement is rare [2]. While scarring is common with MMP involvement at other sites, oral scarring is rare [6]. Disease presentation is variable; some patients are largely asymptomatic or present with mild gingival erythema, while other patients may have widespread oral bullae and ulcers impacting daily activities such as eating and speaking [26]. While the oral cavity is the most common site of involvement (found in 80% of patients), conjunctival involvement is also very common and found in 65% of patients [2]. Other sites of involvement can include the nasal, genital, perianal, and esophageal mucosae [6]. Skin involvement is less common (found in about 25% of cases) and tends to be mild but is associated with residual scar formation, which is particularly noticeable with scalp lesions [14].

Although MMP is not inherently fatal, it can be associated with severe morbidity, particularly when there is involvement of high-risk mucosal sites outside the oral cavity. Ocular involvement is frequent; it often begins as unilateral conjunctival erythema and the sensation of irritation but can evolve into progressive scarring and fibrosis, resulting in entropion (lids turning inwards, causing lashes to rub against the cornea), which can ultimately result in long-term vision loss or blindness [2,26]. Lesions can also cause esophageal strictures, leading to dysphagia and possible weight loss [27]. Finally, although rare, MMP can lead to laryngeal stenosis, causing life-threatening outcomes like airway obstruction [14,28]. Therefore, a thorough review of systems should guide referrals for assessment of potentially involved high-risk mucosal surfaces in order to adequately treat the disease and mitigate adverse outcomes. MMP is rarely associated with an increased risk of cancer, specifically skin cancers and adenocarcinomas. These risks are highest in the first few years after symptom onset, with the greatest risk of skin cancer within the first 5 years and the greatest risk of internal malignancies within the first year [29,30,31,32].

### 3.1. Epidemiology and Pathophysiology

The pathophysiology of MMP is characterized by autoantibodies against hemidesmosomal components of the basement membranes of the epithelium. The most commonly targeted antigens are BP180, BP230, and laminin 332, which are glycoproteins essential for cell adhesion at the dermal–epidermal junction [2,7]. Following production of these autoantibodies, an inflammatory process is initiated, and reactive oxygen species and proteases mediate loss of adhesion, leading to the characteristic blisters and ulcerations seen in MMP [2]. As inflammation recedes and ulcerations heal, progressive fibrosis and scarring typically follow through pathophysiological mechanisms that are not yet fully understood [6].

Epidemiological data for MMP is very limited given the disease’s rarity; however, one study from Finland estimated the incidence of MMP to be between 1.3 and 2.0 per million per year [7]. Females are about twice as likely to have MMP as males [14]. The onset of MMP is typically in the middle and later years of life, with patients most often being diagnosed between 60 and 80 years old [7]. No ethnic or geographic associations have been found [2]. Studies have identified genetic components of MMP; while MMP does not appear to be inherited in a Mendelian pattern, it is associated with certain HLA alleles, such as HLA-DQB1*03, HLA-DRB1*4, and DRB1*11 [2].

MMP may also be triggered by certain drugs. Drug-induced pemphigoid is a well-known phenomenon and is associated with a wide variety of drugs, including antihypertensives with a thiol group like captopril, diuretics like furosemide, and penicillamine [33,34]. Oral lesions typically erupt soon after the patient takes the drug and often involve cutaneous lesions as well [33]. In these cases, the offending drug should be stopped while standard-of-care treatment is initiated.

### 3.2. Diagnosis and Work-Up

The diagnosis of MMP relies on a combination of history, clinical exam findings, histopathology, and immunopathology. As with PV, clinicians should inquire about the evolution of mucosal lesions, including any triggers or exposures prior to the development of blisters. Inquiring about symptoms that may suggest extraoral involvement is also critical in understanding the extent of the disease. Patients should be asked about dysphagia, ocular symptoms, dyspareunia, and cutaneous blisters. Clinicians should also review all medications for possible drugs that can trigger MMP. On physical exam, oral lesions may be clinically indistinguishable from PV and can appear as tense vesicles, ulcerations, and desquamative gingivitis [14]. As with PV, MMP lesions may demonstrate a positive Nikolsky’s sign.

DIF is the gold standard for diagnosis and the key differentiator of MMP from other autoimmune oral diseases like PV, and it reveals linear deposits of IgG and/or IgA and/or C3 at the dermal–epidermal junction [2,35]. Repeated biopsies for DIF increase sensitivity from 70% to 95%; thus, guidelines recommend that biopsies be repeated at least once after a negative result in cases where MMP is clinically suspected [2,35]. IIF can be used to detect circulating autoantibodies, though sensitivity is low (~50%) in MMP; thus, a negative test should not rule out the disease. Microscopy with H & E staining can also be utilized to visualize the sub-epithelial blisters and fibrosis characteristic of MMP, though nondiagnostic features are often seen [2,36].

### 3.3. Treatment

Treatment for MMP varies depending on the severity of the disease. For mild MMP, topical steroids like clobetasol or triamcinolone may be trialed first [37]. If these treatments are ineffective or lesions are widespread, systemic corticosteroids (for short-term control) and immunomodulators are typically added [37]. The most commonly used immunomodulators for mild MMP are dapsone, tetracyclines, and methotrexate. If the lesions continue to be refractory to treatment, other adjuvant therapies like azathioprine or mycophenolate mofetil may be considered. In very severe cases, cyclophosphamide or corticosteroids can be prescribed concurrently, administered either orally or using IV pulse therapy, though these treatments are used very infrequently and only in the setting of severe cases [2]. Major side effects include the following: (1) risks of chronic steroid use, including immunosuppression, hypertension, muscle atrophy, and osteoporosis; (2) risks of dapsone, including hemolytic anemia and gastrointestinal upset; and (3) risks of cyclophosphamide use, including leukopenia and hemorrhagic cystitis [38]. Given these adverse effects, chronic use of these treatments should be minimized when possible; however, they are effective in promoting a rapid improvement in symptoms and remission in many cases [2].

When symptoms continue to be refractory to these treatments and in the setting of high-risk sites of mucosal involvement, rituximab is often used, either as monotherapy or in combination with other immunosuppressive agents [2]. In one study where patients with severe, refractory MMP received rituximab treatment, patients achieved disease control after about 7 months and complete remission after about 12 months [39]. Rituximab was also effective in maintaining sustained disease control post-treatment, with 69% of patients experiencing complete remission even one year post-treatment [39]. Thus, rituximab is a promising agent for MMP treatment, particularly in refractory or severe cases.

## 4. Oral Lichen Planus

Oral lichen planus (OLP) is an inflammatory disease of the oral mucosa and is one of the most common immune-mediated diseases of the oral cavity [3]. Manifestations in the oral mucosa can be categorized into reticular, erythematous, or erosive/ulcerative forms [3,40]. Lesions often evolve over time in regard to their form, distribution, or severity, and longstanding inflammation can cause the development of chronic mucosal atrophy and fibrosis in some patients [41,42]. These chronic changes can contribute to persistent pain, impaired oral function, and reduced response to conventional therapies, emphasizing the importance of early recognition and treatment.

**Figure 3:**
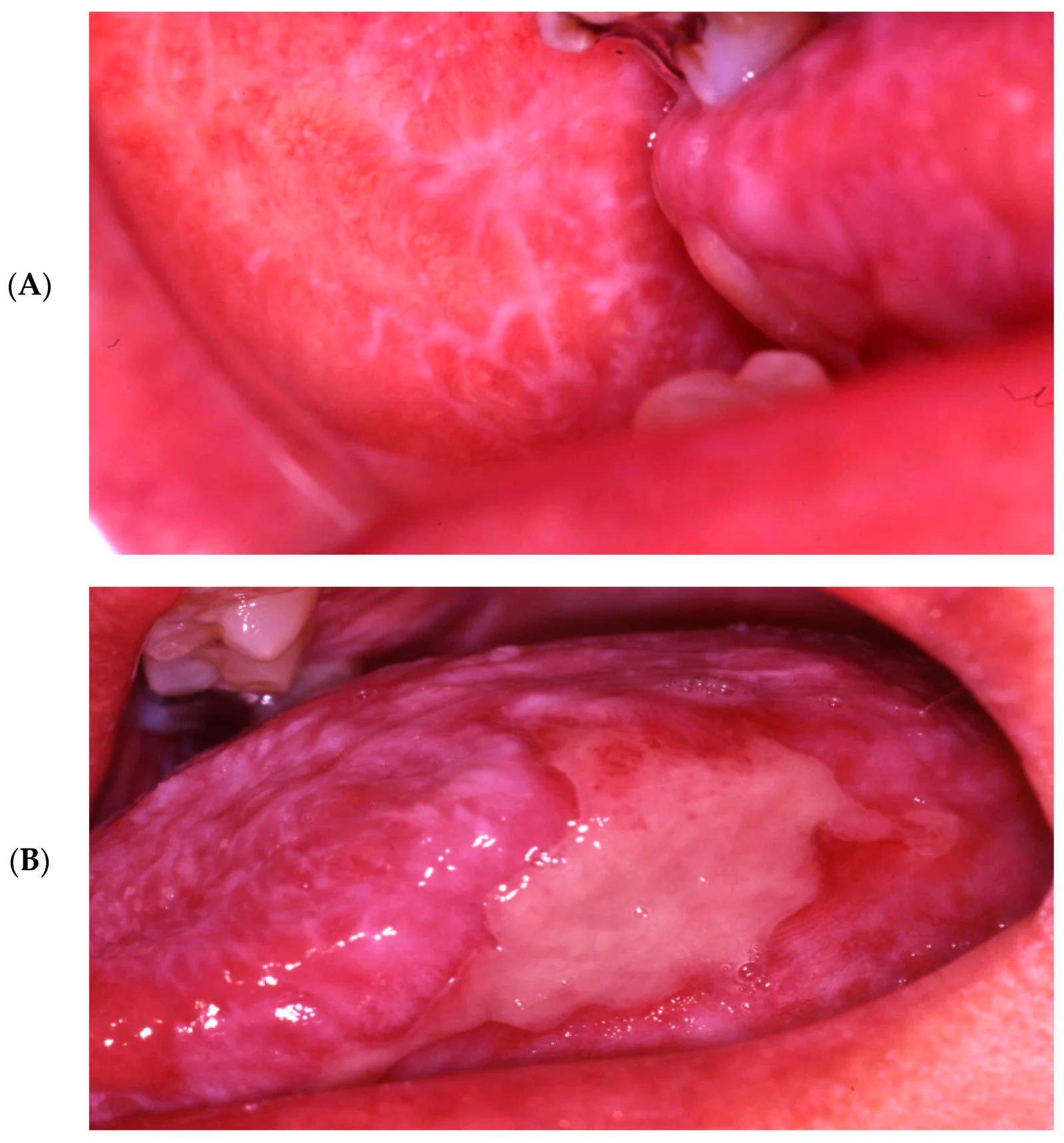
Oral lichen planus. (**A**) Reticular disease with a white lacy, netlike pattern (Wickham’s striae) with a background of erythema on the buccal mucosa. (**B**) Ulceration with an overlying pseudomembrane, as seen in erosive/ulcerative disease of the lateral tongue.

Reticular OLP presents with Wickham’s striae, which are fine white lines that manifest in a web-like pattern on oral mucosa affected by OLP. Wickham’s striae typically present bilaterally and are often found on the buccal mucosa, tongue, or gingiva [43]. In areas where Wickham’s striae are dense, reticular OLP may present as papular or plaque-like with white papules or plaques on the oral mucosa, with the dorsal tongue being a common site of involvement [44,45]. Although reticular OLP is often asymptomatic, it may have associated mild pain or burning [46]. Erythematous OLP presents with erythema and is more often symptomatic, frequently presenting in an overlap with reticular disease (Figure 3A) [47]. Erosive/ulcerative OLP presents as erosions or ulcerations on the oral mucosa that are highly painful (Figure 3B). Bullous OLP is a rare presentation with fluid-filled bullae or vesicles on the oral mucosa [40]. These bullae typically burst and thus precede ulcerative OLP. In addition to oral manifestations, patients may also present with involvement of the genital, esophageal, laryngeal, and conjunctival mucosae, as well as the skin [3,44].

In addition to associated pain, the primary risk of untreated OLP is the potential for malignant transformation to oral squamous cell carcinoma. While OLP has been listed as an oral potentially malignant disorder, there exists some controversy about whether OLP should truly be considered premalignant. Some investigators feel that the presence of epithelial dysplasia is an exclusion criterion for the diagnosis of OLP; however, others argue that OLP can develop epithelial dysplasia during its malignant transformation and that this exclusion criterion would significantly underestimate malignancy associated with OLP [48,49]. Several recent studies have demonstrated a significant association between OLP and oral squamous cell carcinoma and estimated that the risk of malignant transformation is 1–2% [50]. This process tends to be gradual and is associated with chronic, untreated, or undertreated OLP; one study found that the median time until malignant transformation to oral squamous cell carcinoma was 8 years [3,51]. Risk factors for malignant transformation include having the erosive/ulcerative subtype, lesions located on the tongue, older age, and a history of smoking [3,51,52]. Aside from its malignant potential, other risks of OLP may arise from simultaneous extraoral involvement, such as esophageal involvement causing odynophagia and dysphagia, and genital involvement, causing discomfort and possible malignant transformation [53]. Finally, patients can experience malnutrition and weight loss from being unable to tolerate eating, particularly with the erosive/ulcerative subtype.

### 4.1. Epidemiology and Pathophysiology

The precise pathophysiology of OLP is unknown but is suspected to involve an inflammatory process, ultimately leading to keratinocyte death. OLP involves activated CD8+ T-cells interacting with other immune cells, including CD4+ T-cells, macrophages, and Langerhans cells, to trigger apoptosis of oral epithelial cells [3,50].

Aside from immunological dysfunction, several factors are thought to be involved in the onset of OLP. Genetics play a role in OLP; HLAs and polymorphisms in the Vitamin D receptor have been associated with an increased risk of OLP. Several environmental triggers have also been found to trigger the onset of OLP. Dental materials like gold, nickel, and amalgam may trigger a contact sensitivity reaction, leading to the onset of OLP-like lichenoid lesions [45,54,55]. These reactions are particularly important to recognize in older adults, who are more likely to have multiple dental restorations. Oral lichenoid lesions can be clinically distinguished given their direct proximity to dental metals. Unlike idiopathic OLP, management includes identification and removal or replacement of the offending dental material, which may lead to improvement or complete resolution of lesions. Viruses are also hypothesized to be triggers of OLP and include hepatitis C, human papillomavirus, and Epstein–Barr virus [56,57,58,59]. Nutritional deficiencies, such as low Vitamin B12 or D, can be associated with OLP [60]. Imbalances in the gut microbiome and in hormones (particularly in post-menopausal women) have also been associated with increased risk of OLP [3]. Stress is also a well-known trigger of OLP, possibly through creation of a pro-inflammatory state. Finally, dietary triggers, including mint, cinnamon, spicy foods, and acidic foods (citrus fruits, vinegar-based sauces and dressings), have all been well described. Thus, the pathogenesis of OLP is influenced by a multitude of interacting factors that cause disease in susceptible individuals.

In addition to the triggers above, OLP can also be drug-induced. Common drugs that can trigger OLP include beta-blockers, ACE inhibitors, hydrochlorothiazide, NSAIDs, and sulfonylureas [45,61]. The association between the drug and lichenoid mucositis can be difficult to determine given that lesions may become symptomatic months to years later [61]. Discontinuation of the drug should gradually lead to resolution of the symptoms, though standard-of-care treatment of OLP should be initiated [45].

OLP is one of the most common immune-mediated diseases in the oral cavity, affecting about 0.5–2% of the global population [3]. Prevalence is greater in females, with a preference ratio of 2:1 [3]. Further illustrating this point, one study in the United States identified an incidence of OLP in women of 11.4 per 100,000 person-years versus 8.1 in men [62]. Like PV and MMP, OLP tends to begin affecting people who are middle-aged, with an onset often between the ages of 30 and 60; however, in rare cases, OLP has presented in children as well [63,64].

### 4.2. Diagnosis and Work-Up

Diagnosis of OLP requires a combination of clinical history, exam findings, and histopathology. When describing their lesions, patients often report waxing and waning symptoms, with periods of time where their lesions flare up and symptoms are exacerbated. Many patients, particularly those with an erosive subtype, can also identify triggers that worsen their lesions and the associated pain; for instance, spicy, crunchy, and acidic foods are common triggers of OLP flares. When taking a history, clinicians should also inquire about potential triggers of OLP, including recent medication changes, dental work, or a history of smoking. Finally, symptoms that may suggest extraoral involvement should be inquired about, such as the presence of dyspareunia, dysphagia or odynophagia. Physical exam is also a key component of working up OLP. Wickham’s striae significantly increase clinical suspicion of OLP and should prompt clinicians to more thoroughly look for erosions, gingival erythema, or extraoral mucosal involvement. It is also critical to evaluate for possible indications of malignant transformation, including expansion of lesions within the mouth or loss of mucosal homogeneity [50].

Similarly to PV and MMP, the firm diagnosis of OLP relies on biopsy for histopathologic examination and DIF. Laboratory evaluations including CBC, CMP, and hepatitis C serologies may help to determine treatment choice but are not helpful in making the diagnosis [3]. Patients may consider patch testing to rule out possible reactions to dental materials, but only if lesions are localized near dental amalgams [3]. Histopathologic examination of an OLP biopsy typically reveals a characteristic “saw-tooth” appearance of rete ridges from epidermal hyperplasia and hyperkeratosis with lymphocytic infiltration at the dermal–epidermal junction [3,50,65]. Histopathological examination may be helpful in raising clinical suspicion for OLP secondary to drug-induced or hypersensitivity reactions, as these reactions often have increased numbers of eosinophils, while idiopathic OLP has minimal eosinophils [3,65,66]. DIF should also be pursued to differentiate OLP from PV and MMP and often reveals fibrinogen deposition along the basement membrane [61]. Although this finding is not specific to OLP, it can help distinguish it from clinically similar autoimmune oral diseases [65]. IIF is not used to diagnose OLP [65].

### 4.3. Treatment

The goals of treatment for OLP are to control symptoms, reduce inflammation, and minimize the chance of malignant transformation. Treatment is based on patients’ presentation; for asymptomatic patients (particularly those with limited reticular disease), observation may be sufficient, whereas for symptomatic patients (often with the erythematous and erosive/ulcerative subtypes), pharmacological therapy may be warranted.

First-line treatment for OLP includes topical corticosteroids like triamcinolone, fluocinonide, and clobetasol propionate [3,67]. These medications are often available as a paste, gel or ointment and help to decrease inflammation and associated symptoms while avoiding the systemic side effects of oral steroids. However, long-term use of these treatments should be carefully considered, as steroids can predispose patients to oral candidiasis [3]. Similarly, intralesional steroids are often helpful in providing rapid relief of symptoms, particularly in patients with localized erosive/ulcerative disease [3,68]. Patients should also be encouraged to identify and avoid any dietary triggers that worsen their disease. Commonly reported dietary triggers in OLP (as defined as exposures that always or almost always triggered a flare) include spicy foods, raw citrus fruits, mouthwash, toothpaste, crunchy foods, vinegar-based foods, alcohol, and raw non-citrus fruits. In a retrospective study, patients who actively avoided dietary triggers noted improvement in frequency of flares and severity of symptoms [8,69].

In cases of widespread, severe erosive/ulcerative OLP or when symptoms are refractory to topical or intralesional therapy, systemic immunosuppression should be considered. Oral corticosteroids such as prednisone are often one of the most effective treatments, but long-term use should be avoided given the risks of bone demineralization and immunosuppression [68]. Immunomodulatory agents such as metronidazole, hydroxychloroquine, and systemic retinoids, as well as immunosuppressive therapies such as azathioprine and mycophenolate, have demonstrated efficacy in treating OLP [67]. These medications may be particularly useful in situations where patients’ symptoms are refractory to topical corticosteroids and can be used to avoid long-term steroids [60]. Each of these treatments has a unique side-effect profile and should be considered based on individual patients’ risk factors.

Interestingly, emerging evidence also supports Vitamin D as an effective adjunctive treatment for OLP. Vitamin D deficiency has been found to exacerbate OLP symptoms by increasing keratinocyte apoptosis, leading to longer healing times and worse outcomes. Several studies have been conducted on whether Vitamin D supplementation improves OLP outcomes, and in each of these studies, patients who took Vitamin D supplements experienced a significant improvement in their pain and a reduction in the size of their oral lesions [70,71,72,73]. These studies show promise towards better understanding the role that Vitamin D plays in OLP and how vitamin replacement may facilitate improved outcomes for patients.

## 5. Pain Control

Pain is often a source of significant morbidity in patients with PV, MMP, or OLP. Proper pain control is an essential part of initial treatment to help patients maintain their quality of life, nutritional status, and oral hygiene. The options for pain management are broad and are tailored to patients’ unique presentation and disease severity.

### 5.1. Topical Anesthetics and Barrier Treatments

Topical anesthetics are commonly used for rapid, short-term relief of oral pain. The application of lidocaine gel or lidocaine mouth rinses prior to meals can be effective in numbing the oral cavity, helping patients eat without significant pain [2]. However, care should be taken to avoid application to the soft palate and posterior pharynx, as aspiration is a risk. Other agents are also effective in treating pain from oral ulcers, including topical hyaluronic acid, aloe vera gel, benzocaine, and sucralfate, which can form a protective coating over erosions and reduce irritation from mechanical and chemical stimuli [74,75]. Although these agents are effective, they have a limited duration of action and may not be as effective in promoting long-term pain control.

### 5.2. Topical Anti-Inflammatory Treatment

In addition to their disease-modifying effects, topical corticosteroids are highly effective in controlling pain from oral ulcers by reducing local inflammation. Based on the patient’s disease severity, low-potency topical steroids like hydrocortisone can be used; however, higher-potency topical steroids like clobetasol or betamethasone are typically required, especially for severe or refractory disease [75]. These agents can be delivered via a variety of routes, including ointments, gels, and mouth rinses. Topical steroids are preferable to systemic steroids given the reduced side-effect profile; oral candidiasis is the primary adverse effect and can be minimized by concurrent use of antifungals and/or chlorhexidine mouthwash [75]. Chlorhexidine can also be used as an adjunctive treatment in controlling pain from oral ulcers via its anti-inflammatory effects, though discoloration of teeth with this agent should be taken into consideration [76].

### 5.3. Systemic Analgesics

Systemic analgesics can be utilized alongside the methods above to help control pain. Milder analgesics like acetaminophen or NSAIDs can be used for mild pain; however, patients’ co-morbidities should be carefully considered before prescribing NSAIDs for an extended duration of time. NSAIDs have been implicated in drug-induced OLP, so caution should be taken in that patient population [77]. In more severe cases where patients present with debilitating pain, particularly during acute flares or uncontrolled disease, temporary use of opioids may be considered.

### 5.4. Irritants to Avoid

Lifestyle changes are also central in controlling pain from these oral autoimmune diseases. Patients should carefully monitor which foods and drinks cause their symptoms to acutely worsen. Common triggers include acidic, spicy, hot, vinegar-based, and crunchy foods, which can cause both mechanical and chemical irritation to inflamed mucosa. Other irritants include tobacco or alcohol use, as well as certain toothpastes and mouthwashes with mint or cinnamon flavors [8]. These irritants are most commonly associated with oral ulcer flare-ups; however, because triggers vary widely between patients, patients should be encouraged to keep track of their personal triggers and suitable alternatives.

## 6. Oral Hygiene

Because of the painful nature of these diseases, it can be difficult for patients to maintain adequate oral hygiene. However, supporting patients in achieving consistent oral care is essential to reducing secondary infections, minimizing plaque-induced inflammation, and promoting the overall health of oral mucosa. In addition to discussing the pain control options above, patients should be provided with education on lifestyle modifications to improve comfort and effectiveness while engaging in oral hygiene.

Patients should be advised to use soft-bristled toothbrushes and soft dental floss or water flossers to minimize mechanical trauma to inflamed or eroded mucosa [2]. Small-headed brushes may also improve access while allowing for more controlled movements. Brushing techniques should be gentle to avoid irritating the oral mucosa, and patients may need to temporarily use methods like foam swabs during severe disease flare-ups if the pain is not tolerable.

Selecting oral care products is also an important consideration. Toothpastes and mouthwashes free of common irritants, particularly mint- and cinnamon-flavored agents, are recommended, as these additives can exacerbate mucosal irritation [8]. Alcohol-free mouthwashes are also preferred to avoid further irritating the oral mucosa. Bland rinses, such as saline solutions, can also be used to provide symptomatic relief and decrease inflammation. Sodium laurel sulfate-containing products should be avoided.

Importantly, patients should be counseled to maintain routine visits with dental providers, which allow for professional cleaning, monitoring of oral disease activity, early identification of complications like secondary infections, and support by identifying a comfortable and effective oral hygiene regimen. Treatment may need to be modified prior to and following a dental procedure in anticipation of a disease flare-up, and close collaboration between dental and medical providers is encouraged to manage these conditions. Some patients find benefit from increased frequency of cleanings, though extra cleanings are often not covered by dental insurance.

The interplay between inflamed gingival tissues due to PV, MMP, or OLP, pain that leads to difficulty maintaining appropriate oral hygiene, and resultant or underlying true periodontal disease is complex. Co-management with a periodontal specialist is helpful for patients with desquamative gingivitis as a clinical presentation of PV, MMP, or OLP.

## 7. Nutritional Considerations

Nutritional management is a key component of holistic care for patients with PV, MMP, or OLP when pain significantly limits oral intake. Inadequate nutrition may lead to weight loss, delayed mucosal healing, increased susceptibility to infection, and overall decline in health and quality of life. Therefore, it is essential to monitor patients’ nutritional status and counsel them on ways to maintain their nutrition even in the midst of painful disease.

Patients should be encouraged to identify diets that minimize irritation to their oral mucosa. Consuming soft, pureed, or liquid foods is often better tolerated by patients since these do not cause significant trauma to the fragile mucosa. Additionally, patients should be counseled on chemical irritants in food, including acidic, spicy, and salty foods, which can exacerbate pain and inflammation. Hot foods and drinks, as well as carbonated drinks, should also be minimized to avoid mucosal irritation. To maintain adequate caloric intake, patients may benefit from small, frequent meals, as well as the incorporation of calorie-dense, nutrient-rich options like smoothies and protein shakes. If patients experience weight loss or persistent difficulty identifying an appropriate diet, referral to a dietician could be considered for individualized nutritional planning.

Weight loss is a particularly concerning finding that should be monitored. Due to severe pain, patients may significantly decrease their caloric intake, leading to weight loss. In severe cases, extraoral spread of PV, MMP, or OLP can also lead to dysphagia, making it difficult for patients to consume enough calories. In cases of suspected esophageal involvement, referral to gastroenterology should be considered to further work up and treat patients’ dysphagia and monitor their nutritional status.

## 8. Mental Health

Patients with autoimmune blistering disorders face high rates of mental health co-morbidities due to chronic pain and the challenge of living with a relatively rare disease. For instance, the odds of having depression and anxiety in patients with MMP were 1.98 times and 2.26 times higher respectively than those of the general population [7]. Similarly, several studies have found a statistically significant increase in depression and anxiety rates among patients with PV compared to the general population [9,78,79,80]. These data highlight the importance of screening for mental health conditions in patients with autoimmune oral disorders and ensuring that they are holistically supported and connected with mental health providers, as needed.

In addition to the development of mental health co-morbidities, the stress associated with these autoimmune disorders also plays a large role in the presentation of these diseases. For instance, stress is a major trigger of the onset and flare-up of OLP. One study found that 63.2% of patients with OLP reported a stressful life event at the onset of their diagnosis, and another study found that 77% of participants reported stress as a trigger of their OLP flare-ups [8,10]. Given the intricate connection between stress and the manifestation of these diseases, it is critical that providers support patients’ mental health while treating their primary condition.

## 9. Referral Considerations

Once PV, MMP, or OLP is suspected in a primary care setting, timely referral to appropriate specialists is essential to facilitate prompt work-up and treatment. Given the often multisystemic involvement and potential complications of these conditions, a multidisciplinary approach is often required.

### 9.1. Dermatology

Referral to dermatology is an important first step for all patients with suspected PV, MMP, or OLP. Dermatologists play a key role in confirming the diagnosis through clinical evaluation and diagnostic studies, which provide necessary information to distinguish between autoimmune blistering disorders. Dermatologists can also facilitate appropriate treatment, including initiating and monitoring immunosuppressive treatments. Given that these diseases are often underdiagnosed and are associated with severe morbidity, early referral to dermatology to begin the work-up and treatment process is essential.

### 9.2. Dentistry

Referral to dental providers is another key part of holistic care for patients with autoimmune oral disorders. As described above, these providers play a key role in optimizing oral hygiene strategies, monitoring disease progression, and managing oral health complications for patients. Regular dental follow-up is especially important in patients with chronic erosive disease, in whom surveillance for malignant transformation is needed.

### 9.3. Ophthalmology

Ophthalmology referral is highly valuable in assessing ocular involvement. In particular, ophthalmologists play a key role in managing MMP with ocular involvement, which may lead to conjunctival scarring or vision loss if untreated. Although patients with MMP are at increased risk of ocular involvement, all patients with PV, MMP, or OLP should receive ophthalmological evaluation, particularly when treated with corticosteroids, which can lead to long-term ocular complications like glaucoma. Therefore, patients benefit from a baseline ophthalmological evaluation, followed by routine follow-up to monitor for the development of new symptoms.

### 9.4. Gastroenterology

PV, MMP, and OLP can all present with esophageal involvement and can cause erosions or strictures in the esophageal mucosa. Gastroenterology (GI) referral should be considered for patients with suspected esophageal involvement, indicated by symptoms like dysphagia, odynophagia, reflux, or unexplained weight loss. If these symptoms are identified, prompt referral to gastroenterology is critical to avoid further weight loss or nutritional compromise.

## 10. Special Considerations for Geriatrics

Given that PV, MMP, and OLP often present in older populations, special consideration needs to be given to factors that affect this vulnerable population [81].

Aging is associated with significant changes that impact oral health, which can complicate the presentation and recognition of PV, MMP, and OLP in older adults. Older adults commonly experience xerostomia due to age-related salivary gland dysfunction and medication use, as well as periodontal disease, dental caries, poorly fitting dentures, and oral candidiasis [82]. These conditions may mimic or obscure symptoms of autoimmune oral disease, contributing to diagnostic delays. In addition, older patients may attribute oral discomfort to normal aging or dental problems and therefore may present later in the disease course. Distinguishing between autoimmune processes and other age-associated conditions affecting the oral health of older patients is key in timely diagnosis and treatment.

Frailty is another important consideration when managing PV, MMP, and OLP in older adults. Painful oral lesions can impair oral intake, resulting in possible weight loss, sarcopenia, dehydration, malnutrition, and functional decline [2,11]. These consequences may be particularly severe in older adults with high frailty, who have limited physiologic reserve and are at increased risk of hospitalization, falls, and loss of independence. Routine assessment of weight, nutritional status, muscle strength, and functional capacity should therefore be incorporated into clinical care. Early referral to nutrition services may also help prevent progression of malnutrition and frailty.

Another concern related to frailty is the adverse effects of corticosteroids, particularly on older adults. As previously discussed, corticosteroids may contribute to hypertension, hyperglycemia, osteoporosis, and increased risk of infection [12]. Age-related declines in renal and hepatic function may further alter medication metabolism and increase toxicity risk. Older adults are at heightened risk of morbidity from these effects, and corticosteroid use should be carefully monitored in this population to support the lowest effective dose and transition to non-steroidal treatments, if possible. Preventive measures, such as bone scans or glucose checks, should be implemented to screen for adverse effects of these treatments.

Polypharmacy is another key consideration when treating older patients. Patients often have extensive treatment regimens for co-morbidities, leading to potential drug interactions, adverse effects, or missed doses. Polypharmacy can increase confusion regarding treatment regimens for patients, leading to difficulty managing medications and possible nonadherence. Clinicians should conduct careful medication reconciliation to eliminate unnecessary medications and minimize adverse effects, as well as use clear written and visual instructions to support patients’ understanding of their treatment regimens.

Social barriers to care should also be considered when working with older populations. Older patients often face challenges in seeking care, including difficulty obtaining transportation, limited mobility, and financial constraints. Furthermore, social isolation is a major cause of morbidity in older populations, which can reduce support for obtaining care or managing medications, as well as increase the risk of mental health co-morbidities. Careful screenings are critical for early identification of social barriers, allowing for targeted interventions like the involvement of social workers, care coordination services, and connection to community resources to support access to care and overall health outcomes.

Finally, management goals should remain patient-centered. In patients with significant frailty, advanced co-morbidities, or limited life expectancy, patients may choose to prioritize symptom control, oral intake, functional status, and quality of life over complete disease remission. Shared decision-making involving patients, caregivers, and providers is essential to ensure that treatment plans align with individual goals and preferences.

## 11. Clinical Pearls and Key Takeaways

Work-UpChronic, painful oral lesions warrant further evaluation, especially if they are not responsive to common treatments.Persistent oral ulcers, erosions, or gingival inflammation lasting more than 2–3 weeks (particularly those unresponsive to standard therapies) should raise concern for an underlying autoimmune mucosal disorder.Desquamative gingivitis is a concerning finding for an autoimmune condition.Diffuse erythema, sloughing, or bleeding of the gingiva should prompt consideration of mucous membrane pemphigoid, oral lichen planus, or pemphigus vulgaris, even if discrete erosions are not present.Pain out of proportion to exam can indicate a possible autoimmune process.Patients may report severe discomfort despite subtle visible findings, especially in early disease or after bullae have ruptured.Screen for multisite involvement, particularly if an autoimmune process is suspected.Ask about dysphagia, hoarseness, ocular symptoms, or genital lesions, which may indicate more extensive disease and need for additional referrals.ManagementEarly dermatology referral is critical.Definitive diagnosis requires biopsy with direct immunofluorescence; timely referral to dermatology can prevent delays in initiating disease-modifying therapy.Initiate supportive care while awaiting specialty evaluation.Topical corticosteroids, pain control measures, gentle oral hygiene practices, and dietary modifications can significantly improve symptoms and quality of life.Screen for nutritional compromise and mental health impacts.Weight loss and reduced oral intake are common, particularly in older adults, and may require early intervention or dietitian referral. Mental health co-morbidities like anxiety and depression are also common; frequent screenings should be conducted and may require referral to mental health support.Carefully monitor patients receiving treatment for medication-related adverse effects.Systemic corticosteroids and immunosuppressive therapies carry increased risks in geriatric patients; patients should be carefully monitored for development of adverse effects in partnership with the prescribing physician.Be mindful of factors that disproportionately affect older patients’ access to care.Older adults face higher rates of frailty, polypharmacy, and social barriers; patients should be carefully screened for these barriers and supported through medication reconciliation, clear patient education, and connection to supportive resources.Encourage regular dental follow-up.Dental providers play a key role in monitoring disease progression, supporting oral hygiene, identifying complications, and reinforcing patient education.

## 12. Conclusions

Autoimmune conditions affecting the oral mucosa, including PV, MMP, and OLP, are a significant cause of morbidity in older adults. These conditions are often underdiagnosed, and many patients suffer for years before receiving a diagnosis and treatment. Early recognition in the primary care setting and timely referral are critical to properly supporting these patients, allowing for diagnostic confirmation, initiation of appropriate treatment, and prevention of disease-related complications. Effective treatment requires a multidisciplinary approach that focuses not only on disease management but also on pain control, oral hygiene, nutrition, and mental health to support patients’ quality of life and overall functioning. Special considerations should be given to older adults, as these patients are more vulnerable to factors like frailty, polypharmacy, and social barriers that may complicate management. Through this comprehensive, patient-centered approach, clinicians can holistically support older patients throughout the disease course, ultimately improving clinical outcomes, preserving functional status, and enhancing overall quality of life.

## Data Availability

No new data were created or analyzed in this study. Data sharing is not applicable to this article.
